# Supplementary material for: Content-rich biological network constructed by mining PubMed abstracts
Source: BMC Bioinformatics. 2004 Oct 8;5:147. doi: 10.1186/1471-2105-5-147 (PMC528731; doi:10.1186/1471-2105-5-147)
Supplement: Additional File 2 — The original results of the above study (non-essential files are deleted to keep the file size under the limit set by BMC bioinformatics). [file 1471-2105-5-147-S2.bz2 › chilibotAdditionalFile2/dip50/329ID2158863E3912/html/TNFRSF1A_TNF.html]

 


 **TNFRSF1A** and **TNF** 
  
Found 1721 abstracts in PubMed, retrieved 50.  
 

 What does Google say? 
 PDF only 
| .edu only 

---

**Interactive relationship** (e.g. stimulation, inhibition, etc)

**Stimulatory relationship**- In addition, NF kappaB downstream of TNFR  [ **TNFRSF1A** ]  was required for upregulation of Fas expression by endogenous  **TNF**  secreted in response to TCR stimulation.  Ref: 12813034 J Biol Chem< MedlineTA>J Biol Chem,
- TNFR  [ **TNFRSF1A** ]  upregulation in injured and adjacent uninjured DRG neurons may be essential for mediating enhanced  **TNF**  effects and thus contribute to the development of pain related behavior.  Ref: 12875915 Neurosci Lett, 2003
- RESULTS Although non MMF based immunosuppression in ST patients did not result in compromised AC or lipopolysaccharide LPS stimulated monokine responses compared with healthy controls, we found MMF therapy to be associated with significantly reduced  **TNF**  R1  [ **TNFRSF1A** ]  expression on monocytes P< ; 0.001, suppressed AC P< ; 0.02, SED, and suppressed LPS stimulated IL 1beta, IL 10, and  **TNF**  alpha  [ **TNF** ]  secretion P< ; 0.01.  Ref: 12829918 Transplantation< MedlineTA>Transplantation, 2003
**Neutral relationship**- LT consists of soluble LT alpha 3, which binds to  **TNF**  R1  [ **TNFRSF1A** ]  and  **TNF**  R2, and membrane LT alpha 1 beta 2, which binds to LT beta R.  Ref: 12882833 Int Immunol, 2003
- The upregulation of TNFR2 but not TNFR1  [ **TNFRSF1A** ]  was blunted in  **TNF**  alpha  [ **TNF** ]  deficient mice indicating ligand dependent upregulation of TNFR2.  Ref: 12865254 Am J Physiol Renal PhysiolAm J Physiol Renal Physiol, 2003
- Nuclear factor kappaB inducing kinase NIK is a member of the MAP kinase kinase kinase family that was first identified as a component of the  **TNF**  R1  [ **TNFRSF1A** ]  induced NF kappaB activation pathway  **TNF** , tumor necrosis factor  [ **TNF** ] .  Ref: 12853971 Oncogene, 2003
- As the  **TNF**  and TNFR  [ **TNFRSF1A** ]  superfamilies have grown to more than two dozen combined members over the past 30 years, their involvement in interactions between immune cells, with regard to the events governing cellular differentiation, activation, and survival have been well established.  Ref: 12787563 Cytokine Growth Factor Rev, 2003
- Both  **TNF**  receptors were detected, with  **TNF**  R1  [ **TNFRSF1A** ]  being prominent on bronchial epithelial cells and endothelial cells, and  **TNF**  R2 being expressed by nearly all cell types.  Ref: 12849708 Cytokine, 2003
- Western blotting against  **TNF**  RI  [ **TNFRSF1A** ]  revealed two bands of lower molecular weight in cachectic muscle, suggesting proteolysis involving  **TNF**  RI  [ **TNFRSF1A** ] .  Ref: 12851692 Int J Oncol, 2003
- Moreover, renal  **TNF**  alpha  [ **TNF** ]  expression, ICAM 1 expression and serum  **TNF**  alpha  [ **TNF** ]  levels were lower in TNFR2 deficient mice compared with wild type or TNFR1  [ **TNFRSF1A** ]  deficient mice treated with cisplatin.  Ref: 12865254 Am J Physiol Renal PhysiolAm J Physiol Renal Physiol, 2003

**Non-interactive relationship** (e.g. studied together, co-existance, homology, etc.)

- It turned out that antibodies to  **TNF**  R1  [ **TNFRSF1A** ] , but not to  **TNF**  R2 reduced hyperalgesia, indicating that  **TNF**  R1  [ **TNFRSF1A** ]  is the receptor concerned.  Ref: 12799919 Schmerz, 1999
- **TNF**  RI  [ **TNFRSF1A** ]  protein levels confirmed up regulation in the spleen and liver, but failed to detect any increase in the muscle.  Ref: 12851692 Int J Oncol, 2003
- Coinciding with a significantly higher steroid dosage in CR patients, IL 6 receptor and  **TNF**  R1  [ **TNFRSF1A** ]  expression on monocytes were down regulated P< ; =0.02 and AC was suppressed in CR compared with ST non MMF patients P< ; 0.01, SED.  Ref: 12829918 Transplantation< MedlineTA>Transplantation, 2003
- **TNF**  RI  [ **TNFRSF1A** ]  mRNA was up regulated in the spleen, liver and muscle of tumor bearing rats.  Ref: 12851692 Int J Oncol, 2003
- RESULTS Severity of CIA was significantly decreased in  **TNF**  R  [ **TNFRSF1A** ]  treated animals compared with controls, 14 34 days after disease onset.  Ref: 12860724 Ann Rheum DisAnn Rheum Dis, 2003
- Two closely related variants, TNFalpha and TNFbeta, elicit various cellular responses via two distinct  **TNF**  receptors, the 55 kDa  **TNF**  R1  [ **TNFRSF1A** ]  and the 75 kDa  **TNF**  R2.  Ref: 12849708 Cytokine, 2003
- We have previously reported the pattern of cellular expression of tumor necrosis factor  [ **TNF** ]  receptors TNFR  [ **TNFRSF1A** ]  in human kidney and their altered expression in transplant rejection.  Ref: 12875962 Am J Pathol, 2003
- **TNF**  R1  [ **TNFRSF1A** ]  p55 serves as the principal receptor responsible for MCP 1 synthesis.  Ref: 12838504 Glia, 2003
- The fusion of the transmembrane domain of LMP1 to signaling domains of CD40,  **TNF**  R1  [ **TNFRSF1A** ]  and Fas activates their signaling.  Ref: 12805217 EMBO J, 2003
- This activity of LT depends on membrane LT alpha 1 beta 2 and not soluble LT alpha 3, since DA virus persisted and induced inflammatory demyelination in LT beta R, but not  **TNF**  R1  [ **TNFRSF1A** ]  or  **TNF**  R2, mice.  Ref: 12882833 Int Immunol, 2003
- Earlier study showed that glucocorticoid induced tumor necrosis factor  [ **TNF** ]  receptor GITR, a new TNFR  [ **TNFRSF1A** ]  family, activated murine macrophages to express inducible nitric oxide synthase iNOS and to generate nitric oxide NO.  Ref: 12858016 Exp Mol Med, 2003
- Pretreatment with soluble recombinant  **TNF**  receptor TNFR  [ **TNFRSF1A** ]  Fc, 10 & ; mgr ; g kg 1, i.c.  Ref: 12787340 Neurogastroenterol Motil< MedlineTA>Neurogastroenterol Motil, 2003
- Joint destruction was reduced in  **TNF**  R  [ **TNFRSF1A** ]  injected joints and in the uninjected contralateral and ipsilateral paws of  **TNF**  R  [ **TNFRSF1A** ]  treated animals.  Ref: 12860724 Ann Rheum DisAnn Rheum Dis, 2003
- The common neurotrophin receptor p75 NTR remains one of the most enigmatic of the tumor necrosis factor  [ **TNF** ]  receptor TNFR  [ **TNFRSF1A** ]  superfamily on the one hand, it displays a death domain and has been shown to be capable of mediating programmed cell death PCD upon ligand binding.  Ref: 12787561 Cytokine Growth Factor Rev, 2003
- We analyzed expression of  **TNF**  alpha  [ **TNF** ] , IL 6, tumor necrosis factor  [ **TNF** ]   **TNF**  RI  [ **TNFRSF1A** ] ,  **TNF**  RII and interleukin 6 IL 6R receptors in the brain, kidney, spleen, liver, muscle, ascite tumors and serum, from Yoshida AH 130 hepatoma bearing rats.  Ref: 12851692 Int J Oncol, 2003
- cFLIP down regulation induced by cFLIP antisense oligodeoxynucleotides sensitized HLE cells to Fas,  **TNF**  R  [ **TNFRSF1A** ] , and TRAIL R mediated apoptosis.  Ref: 12861043 Lab Invest, 2003
- Immunity to Toxoplasma gondii critically depends on TNFR  [ **TNFRSF1A** ]  type I mediated immune reactions, but the precise role of the individual ligands of TNFR1  [ **TNFRSF1A** ] ,  **TNF**  and lymphotoxin alpha LTalpha, is still unknown.  Ref: 12794148 J Immunol< MedlineTA>J Immunol, 2003
- The difference in  **TNF**  R  [ **TNFRSF1A** ]  II levels between seropositives and AIDS was however more impressive.  Ref: 12815299 Methods Cell Sci, 2002
- Neutralizing anti  **TNF**  R1  [ **TNFRSF1A** ]  antibodies inhibited macrophage activation assessed by Fas L expression and NO secretion.  Ref: 12869351 Arterioscler Thromb Vasc BiolArterioscler Thromb Vasc Biol, 2002
- Additionally, fusion constructs of the transmembrane domain of LMP1 and the intracellular signaling domain of CD40,  **TNF**  R1  [ **TNFRSF1A** ] , and  **TNF**  R2 likewise transactivated the CD83 promoter via NF kappaB.  Ref: 12857898 J VirolJ Virol, 2002
- The Fas associated death domain FADD adaptor protein FADD Mort 1 is recruited by several members of the tumor necrosis factor  [ **TNF** ]  receptor TNFR  [ **TNFRSF1A** ]  superfamily during cell death activated via death receptors.  Ref: 12815462 Cell Death Differ, 2003
- The p75 neurotrophin receptor p75NTR is a member of the tumor necrosis factor  [ **TNF** ]  receptor TNFR  [ **TNFRSF1A** ]  superfamily, and can mediate both cell survival and cell death in response to nerve growth factor NGF.  Ref: 12852261 Vitam Horm, 2003
- The overall influence of  **TNF**  R  [ **TNFRSF1A** ]  gene therapy is that it inhibits the progression of CIA mainly by suppressing the inflammatory Th1 response rather than by stimulating a Th2 response.  Ref: 12860724 Ann Rheum DisAnn Rheum Dis, 2003
- The tumor necrosis factor  [ **TNF** ]  receptor 1  [ **TNFRSF1A** ]  TNFR1  [ **TNFRSF1A** ] , a prototypic member of the death receptor family signals both cell survival and apoptosis.  Ref: 12887914 Cell, 2003
- Tumor Necrosis Factor  [ **TNF** ]  Receptor 1  [ **TNFRSF1A** ]  and 2 Double Deficiency Reduces Graft Arterial Disease in Murine Cardiac Allografts.  Ref: 12859531 Am J TransplantAm J Transplant, 2003
- We investigated whether this particular SNP, and also one at position 36 in exon 1 of the  **TNF**  receptor I  **TNFRSF1A**  gene, are associated with disease severity.  Ref: 12858434 J Rheumatol, 2003
- We tested the hypothesis that activation of specific apoptotic pathways represents a mechanism for tubular cell death after CAD kidney transplantation.METHODS Serial tissue sections from paraffin embedded needle biopsy specimens obtained at approximately 1 hr of reperfusion after transplantation of 13 CAD and 12 living related donor LRD renal allografts were examined by using the terminal deoxynucleotide transferase mediated dUTP nick end labeling assay to detect apoptosis and by immunohistochemistry for expression of key pro apoptotic molecules Bax, Bak, tumor necrosis factor  [ **TNF** ]  receptor TNFR 1  [ **TNFRSF1A** ] , Fas, and cytochrome c.  Ref: 12865785 Transplantation, 2003
- TweakR Fn14 is the smallest member of the  **TNF**  receptor TNFR  [ **TNFRSF1A** ]  superfamily described to date, and it appears to signal via recruitment of several different TNFR  [ **TNFRSF1A** ]  associated factors.  Ref: 12787562 Cytokine Growth Factor Rev, 2003
- Gene knockout study suggests that NIK is dispensable for  **TNF**  R1  [ **TNFRSF1A** ]  but required for lymphotoxin beta receptor induced NF kappaB activation.  Ref: 12853971 Oncogene, 2003
- Fas CD95 or APO 1 is a member of tumor necrosis factor  [ **TNF** ]  receptor TNFR  [ **TNFRSF1A** ]  family.  Ref: 12806430 Drug News Perspect, 2001
- We correlated pain behavior in response to mechanical stimulation with immunoreactivity for  **TNF**  receptor TNFR 1  [ **TNFRSF1A** ]  and 2 at 6 and 120 h following L5 and L6 spinal nerve ligation SNL.  Ref: 12875915 Neurosci Lett, 2003
- Antibody inhibition of caspase 3 activity as well as flow cytometry experiments implicated  **TNF**  related apoptosis inducing ligand TRAIL and  **TNF**  alpha  [ **TNF** ]  receptor  **TNF**  R  [ **TNFRSF1A** ]  in apoptosis signaling.  Ref: 12842625 Virology, 2003
- In situ localization of TNFalpha beta, TACE AND  **TNF**  receptors  **TNF**  R1  [ **TNFRSF1A** ]  and  **TNF**  R2 in control and LPS treated lung tissue.  Ref: 12849708 Cytokine, 2003
- There is extensive crosstalk between the apoptotic and NF kappaB signaling pathways that emanate from  **TNF**  R1  [ **TNFRSF1A** ] .  Ref: 12824002 Cytokine< MedlineTA>Cytokine, 2003
- METHODS Arthritic DBA 1 mice received single periarticular injections of retroviral constructs encoding human  **TNF**  receptor  **TNF**  R  [ **TNFRSF1A** ]  into the affected arthritic paw, at the onset of arthritis.  Ref: 12860724 Ann Rheum DisAnn Rheum Dis, 2003
- In the present study in normal rat and human lung tissue, the constitutive expression of TNFalpha beta, TACE and  **TNF**  R1  [ **TNFRSF1A** ]  R2 was investigated by immunohistochemical techniques.  Ref: 12849708 Cytokine, 2003
- The genes involved in these syndromes are respectively  **TNFRSF1A**   **TNF**  receptor 1 and CIAS1 a gene containing both pyrin and NBS domains.  Ref: 12832748 J Nephrol< MedlineTA>J Nephrol, 2003
- Our results imply that members of the  **TNF**  R  [ **TNFRSF1A** ]  family can signal from intracellular compartments containing lipid rafts and may do so when they act in autocrine loops.  Ref: 12805217 EMBO J, 2003
- As several pathways are involved in induction of apoptosis of T cells, including transforming growth factor TGF beta TGF receptor TGFR,  **TNF**  alpha  [ **TNF** ]  TNFR1  [ **TNFRSF1A** ] , and Fas Fas ligand, these mediators were also investigated in peripheral blood samples from these subject groups.  Ref: 12851215 Am J Physiol Lung Cell Mol Physiol< MedlineTA>Am J Physiol Lung Cell Mol Physiol, 2003
- As  **TNF**  exerts its effects via two receptors,  **TNF**  receptor 1  **TNF**  R1  [ **TNFRSF1A** ]  and  **TNF**  receptor 2  **TNF**  R2, we also investigated, which of the receptors is relevant to the causation of pain in this model.  Ref: 12799919 Schmerz, 1999
- It is concluded that a combination of absolute number of CD4 cells and  **TNF**  R  [ **TNFRSF1A** ]  II assay along with clinical evaluation may be used to monitor therapy in resource poor countries where frequent viral load assay is unaffordable.  Ref: 12815299 Methods Cell Sci, 2002
- This presentation summarizes the roles of TNFR  [ **TNFRSF1A** ]  and  **TNF**  like cytokines in infection, sepsis and autoimmunity as well as the pivotal involvement of these molecules in the development of secondary lymphoid organs.  Ref: 12787558 Cytokine Growth Factor Rev, 2003
- ... MMF treatment might inhibit chronic graft rejection because of suppression of  **TNF**  R1  [ **TNFRSF1A** ]  expression and vigorous inhibition of monokine secretion.  Ref: 12829918 Transplantation< MedlineTA>Transplantation, 2003
- Because cellular FLICE caspase 8 inhibitory protein cFLIP is a recently identified intracellular inhibitor of caspase 8 activation that potently inhibits death signaling mediated by all known death receptors, including Fas,  **TNF**  receptor  **TNF**  R  [ **TNFRSF1A** ] , and  **TNF**  related apoptosis inducing ligand receptors TRAIL Rs, we investigated the expression and function of cFLIP in human HCCs.  Ref: 12861043 Lab Invest, 2003
- We used  **TNF**  type 1 receptor deficient TNFR1KO, type 2 receptor deficient TNFR2KO and receptor double deficient TNFRDKO mice to assess the relative roles of TNFR  [ **TNFRSF1A** ]  in acute rejection and GAD.  Ref: 12859531 Am J TransplantAm J Transplant, 2003
- In the present studies, p55  **TNF**  receptor 1 TNFR1  [ **TNFRSF1A** ]  knockout mice were used to assess the role of  **TNF**  alpha  [ **TNF** ]  in acetaminophen induced antioxidant defense.  Ref: 12842828 Am J Physiol Gastrointest Liver Physiol< MedlineTA>Am J Physiol Gastrointest Liver Physiol, 2003
- By examining surface expression of numerous candidate molecules tumor necrosis factor  [ **TNF** ]  receptor TNFR 1  [ **TNFRSF1A** ] , TNFR2, CD36, CD40, CD47, CD80, CD86, PD L1, CD95 ligand, and type I interferon receptor, we report that IPE cells uniquely express on their surface the costimulatory molecule CD86.  Ref: 12835481 J Exp Med, 2003
- Seven days after disease onset,  **TNF**  R  [ **TNFRSF1A** ]  treated mice had lower levels of inflammatory Th1 driven IgG2a antibodies to CII p< ; 0.05 than controls.  Ref: 12860724 Ann Rheum DisAnn Rheum Dis, 2003
- LMP1 associates simultaneously with lipid rafts and with its signaling molecules, tumor necrosis factor  [ **TNF** ]  receptor  **TNF**  R  [ **TNFRSF1A** ]  associated factors TRAFs and  **TNF**  R1  [ **TNFRSF1A** ]  associated death domain protein TRADD intracellularly, although it can be detected at low levels at the plasma membrane, indicating that most of LMP1 s signaling complex resides in intracellular compartments.  Ref: 12805217 EMBO J, 2003
- Expression of TNFR1  [ **TNFRSF1A** ]  gene increased 2.7 fold and expression of Fas gene decreased 3.1 fold compared to intact endosalpinx, which indicates induction of apoptosis triggered by tumor necrosis factor alpha  [ **TNF** ]  and inhibition of the Fas dependent pathway.  Ref: 12802421 Bull Exp Biol Med< MedlineTA>Bull Exp Biol Med, 2003
- Apoptosis induced by  **TNF**  receptor I TNFR1  [ **TNFRSF1A** ]  is thought to proceed via recruitment of the adaptor FADD and caspase 8 to the receptor complex.  Ref: 12887920 Cell, 2003
- Therefore, periarticular  **TNF**  R  [ **TNFRSF1A** ]  gene therapy may have excellent therapeutic potential in RA.  Ref: 12860724 Ann Rheum DisAnn Rheum Dis, 2003
- Two NO donors upregulated VSMC surface  **TNF**  R1  [ **TNFRSF1A** ] , and exogenous  **TNF**  alpha  [ **TNF** ]  induced VSMC apoptosis synergistically with the NO donor diethylenetriamine NO, indicating that NO sensitizes VSMCs to  **TNF**  alpha  [ **TNF** ] .  Ref: 12869351 Arterioscler Thromb Vasc BiolArterioscler Thromb Vasc Biol, 2003
- These data indicate that  **TNF**  alpha  [ **TNF** ]  signaling through TNFR1  [ **TNFRSF1A** ]  plays an important role in regulating the expression of antioxidants in this model.  Ref: 12842828 Am J Physiol Gastrointest Liver Physiol< MedlineTA>Am J Physiol Gastrointest Liver Physiol, 2003
- Male  **Tnf**  receptor I Tnfr  [ **TNFRSF1A** ]  mice and C57BLJ wildtype WT controls were subjected to sham surgery or bile duct ligation.  Ref: 12837754 J Biol Chem< MedlineTA>J Biol Chem, 2003
- This is the first demonstration of a selective up regulation of  **TNF**  RI  [ **TNFRSF1A** ]  in cancer cachexia and suggests that local production of  **TNF**  alpha  [ **TNF** ]  and IL 6 is a corner stone in the induction maintenance of this syndrome.  Ref: 12851692 Int J Oncol, 2003
- Significantly increased apoptosis of PHA stimulated T cells was observed in COPD annexin positive 75.0 14.7% SD vs. control 50.2 21.8% SD, P = 0.006, along with upregulation of  **TNF**  alpha  [ **TNF** ]  TNFR1  [ **TNFRSF1A** ] , Fas, and TGFR.  Ref: 12851215 Am J Physiol Lung Cell Mol Physiol< MedlineTA>Am J Physiol Lung Cell Mol Physiol, 2003
- Antagonistic antibodies to  **TNF**  receptor R1 inhibited VSMC apoptosis, and preincubation of monocytes and VSMCs indicated that  **TNF**  R1  [ **TNFRSF1A** ]  on both cell types contributed to macrophage induced VSMC apoptosis.  Ref: 12869351 Arterioscler Thromb Vasc BiolArterioscler Thromb Vasc Biol, 2003
- Severity of arthritis, antibodies to collagen type II CII, and extent of pathological joint damage of arthritic paws were compared between  **TNF**  R  [ **TNFRSF1A** ]  and media treated control animals, 1 1, and 49 days after disease onset.  Ref: 12860724 Ann Rheum DisAnn Rheum Dis, 2003
- Correspondingly, both monocytes and VSMCs expressed  **TNF**  R1  [ **TNFRSF1A** ] , and macrophages expressed cell surface  **TNF**  alpha  [ **TNF** ] .  Ref: 12869351 Arterioscler Thromb Vasc BiolArterioscler Thromb Vasc Biol, 2003
- Caspase 8 activation is an upstream event in the apoptotic pathway triggered by the activation of cytokines receptors such as  **TNF**  alpha  [ **TNF** ]  receptor 1 TNFR 1  [ **TNFRSF1A** ] , and the presence of caspase 8 activation in fibroblast like cells in the aseptic interface membranes of THAs suggests a possible  **TNF**  alpha  [ **TNF** ]  dependent apoptosis.  Ref: 12834589 Biomaterials, 2003
- CONCLUSIONS Local  **TNF**  R  [ **TNFRSF1A** ]  gene therapy in CIA appears to have systemic effects on the anti CII antibodies.  Ref: 12860724 Ann Rheum DisAnn Rheum Dis, 2003
- Tumor necrosis factor  [ **TNF** ]   **TNF**  and  **TNF**  receptor TNFR  [ **TNFRSF1A** ]  family proteins play important roles in many biological processes.  Ref: 12857975 Immunol Res, 2003
- 8  **TNF**  receptors TNFR  [ **TNFRSF1A** ] .  Ref: 12819136 Genome Res, 2003
- Role of p55 tumor necrosis factor  [ **TNF** ]  receptor 1  [ **TNFRSF1A** ]  in acetaminophen induced antioxidant defense.  Ref: 12842828 Am J Physiol Gastrointest Liver Physiol< MedlineTA>Am J Physiol Gastrointest Liver Physiol, 2003
